# Feed and Host Genetics Drive Microbiome Diversity with Resultant Consequences for Production Traits in Mass-Reared Black Soldier Fly (*Hermetia illucens*) Larvae

**DOI:** 10.3390/insects12121082

**Published:** 2021-12-01

**Authors:** Matthew P. Greenwood, Kelvin L. Hull, Marissa Brink-Hull, Melissa Lloyd, Clint Rhode

**Affiliations:** 1 Department of Genetics, Stellenbosch University, Private Bag X1, Matieland 7602, South Africa; 19395787@sun.ac.za (M.P.G.); 17507537@sun.ac.za (K.L.H.); marissa.brink-hull@uct.ac.za (M.B.-H.); 2 Insect Technology Group Holdings UK Ltd., 1 Farnham Road, Guildford, Surrey GU2 4RG, UK; melissalloyd1993@gmail.com

**Keywords:** microbiome dynamics, black soldier fly, 16S rDNA, next-generation sequencing, mass-rearing, complex trait

## Abstract

**Simple Summary:**

The development of edible insect farming is crucial for meeting agricultural sustainability goals in the face of growing food demands, ongoing depletion of natural resources, and global climate change. The black soldier fly is considered a promising candidate insect for cultivation, with the larvae of this species being capable of converting agri-waste materials into valuable products that can be used as alternatives to conventional, environmentally detrimental animal feedstuffs, such as processed soy and fish commodities. The biodegradative performance of this species is underscored by the activity of intestinal microorganisms, suggesting that knowledge of larval microbial diversity may be valuable for ensuring sustainability and continual improvement of commercial black soldier fly colonies. In this study, the factors that shape the identity and function of larval gut microbial populations were evaluated. It was determined that both host genetics and diet alter the diversity and metabolic potential of intestinal bacteria, with potential downstream effects on economically important larval rearing traits. Therefore, the findings of this study provide a foundation for expanding and exploiting knowledge of the interactions between black soldier flies and their resident microbial communities for optimisation of insect farming practices.

**Abstract:**

Mass rearing the black soldier fly, *Hermetia illucens*, for waste bioremediation and valorisation is gaining traction on a global scale. While the health and productivity of this species are underpinned by associations with microbial taxa, little is known about the factors that govern gut microbiome assembly, function, and contributions towards host phenotypic development in actively feeding larvae. In the present study, a 16S rDNA gene sequencing approach applied to a study system incorporating both feed substrate and genetic variation is used to address this knowledge gap. It is determined that the alpha diversity of larval gut bacterial communities is driven primarily by features of the larval feed substrate, including the diversity of exogenous bacterial populations. Microbiome beta diversity, however, demonstrated patterns of differentiation consistent with an influence of diet, larval genetic background, and a potential interaction between these factors. Moreover, evidence for an association between microbiome structure and the rate of larval fat accumulation was uncovered. Taxonomic enrichment analysis and clustering of putative functional gut profiles further suggested that feed-dependent turnover in microbiome communities is most likely to impact larval characteristics. Taken together, these findings indicate that host–microbiome interactions in this species are complex yet relevant to larval trait emergence.

## 1. Introduction

Insect mass rearing has recently gained traction as an alternative stream for the production of animal feeds and human food. The emergence of this industry responds to a global need to improve sustainability of food systems, while concurrently addressing the rising food demand thresholds of an increasingly large and affluent human population [1]. This particularly affects protein generation systems, where meeting ambitious expansion milestones for livestock, aquaculture, and fishery sectors is challenged by limited resource availability, excessive wastage, and disconcerting future environmental impact projections [2,3,4,5]. In this regard, the rearing of insects affords many opportunities for navigating obstacles that hinder growth of contemporary protein production systems, including greater feed conversion rates, improved spatial efficiency, and a diminished carbon footprint in comparison with many classical livestock species [6,7].

The black soldier fly (BSF), *Hermetia illucens* (Linnaeus, 1758), is a prominent candidate for insect mass rearing [8,9]. The larvae of this species are remarkable degraders of organic material capable of converting large fractions of their feed substrate into recoverable protein (e.g., for the production of animal feeds and human food), lipid (e.g., for use in animal feeds and biofuel production), and chitin (e.g., for the production of textiles, cosmetics and pharmaceuticals) biomass with vast applications in agricultural and biotechnological spheres [10,11,12]. Additionally, the ability of these larvae to consume contaminated organic matter from various levels of the food production chain enables the simultaneous valorisation and bioremediation of waste material within food systems [13,14,15]. It is, therefore, unsurprising that BSF mass-rearing ventures have become increasingly prevalent in recent years [16], with marked improvements in colony management strategies and production rates attributable to ongoing elucidation of rearing optima [17,18,19,20]. However, as with livestock sectors, it is likely that continued growth and sustainability of this nascent industry will rely on the development and integration of advanced molecular technologies, such as genomic monitoring and selection tools, genetic transformation procedures, and other next-generation *omics* data for expediting trait gain and informing managerial decisions [21,22,23,24,25].

It is broadly acknowledged that the diversity of bacteria, archaea, protists, fungi, and viruses harboured by animal gastro-intestinal tracts (GITs) makes notable contributions to host fitness and survivability, primarily through the secretions of enzymes and compounds that adjust gut metabolic function or modulate immune system responses [26,27]. Consequently, the characterisation and manipulation of intestinal microbiota communities in mass-reared insects may permit rapid improvement of biomass accumulation rates and the identification of enzymes with medical, industrial, or other biotechnological significance [16]. Nevertheless, the dynamic nature of gut microbial landscape in many insects may complicate direct implementation of such methodologies, with layered interactions between host immune factors, resident microbial populations, physicochemical properties of the gut, intrinsic properties of the feed, and environmental microbial taxa continually shaping enteric community structure and function [26]. Burgeoning evidence, therefore, endorses consideration of the microbiome as a complex animal phenotype [28,29,30]. By integrating the understanding that other canonical host traits may respond to particular gut communities in a context-dependent manner [31], it becomes apparent that resolving the interplay between host genetic and environmental variation in driving gut microbiota dynamics may be integral to the holistic description of exploitable microbiome–trait relationships.

The inability of BSF to develop on sterile feed substrate indicates that digestive efficiency and growth in this species is dependent on associations with symbiotic microorganisms [32]. It has further been established that supplementing larval diets with specific companion bacteria can influence numerous marketable larval traits, including the efficiency of feed substrate reduction and rates of biomass accumulation [33,34]. Moreover, previous studies have observed phenotypic divergence in parallel with gut microbial community differentiation between BSF larvae [35], suggesting that trait emergence is mediated by features of the broader larval microbiome. However, these putative microbiome-trait relationships have yet to be reproduced and contextualised with respect to drivers of gut microbiome variability that may be relevant to mass-rearing systems. For example, it is already recognised that the choice of larval diet (i.e., environmental variation) plays a significant role in shaping gut microbial communities under both laboratory and mass-rearing conditions [36]. However, there are substantial discrepancies between studies in terms of diversity, structure, and identity of larval GIT communities, even when similar feed substrates are utilised [35,36,37]. This implies that knowledge regarding the feed-related abiotic and biotic drivers of microbiome diversity may be incomplete and that additional deterministic factors could underpin variation in larval gut ecosystems. Given that BSF colonies reared in different closed systems are also likely to be genetically distinct [38,39], it is probable that strain-specific genetic features contribute a significant portion of such between-study disparity in larval microbiome profiles. Therefore, in light of recent suggestions that microbiome-mediated optimisation of BSF rearing stocks is feasible [16], it is important to characterise the interplay between feed and host genetic background in driving microbiome assembly and influencing resultant microbiome-phenotype relationships in BSF larvae.

This study aimed to investigate feed and host genetic background as drivers of BSF larval gut microbiota diversity and simultaneously resolve potential associations between microbiome and larval production traits. In order to address this aim, BSF larvae were reared in a system encompassing both genetic and environmental variation and phenotyped for several economically important production traits, such as final mass, fat content, and protein content. Genetic variation was captured by rearing a recently established captive cohort (F2 generation; referred to as ‘wild’) in parallel with a longstanding, separately domesticated colony (F62 generation; referred to as ‘factory’), while environmental ‘treatments’ were simulated by provision of different industry-relevant feed substrates across the genetic BSF cohorts in a factorial experimental design. Among the larval dietary options, a standard grain-based feed (‘Formulated’), a vegetable waste feed (‘Organic’) and an enriched brewer’s spent grain diet (‘Brewer’s’), together representing a range of nutritional indices, were utilised. An additional low bioburden feed substrate (‘Sterile’), generated by autoclaving the Formulated diet, was included as a control for presence of exogenous bacterial populations in the larval feed substrate. Using a 16S rDNA bacterial community profiling approach, the BSF larval gut microbiome was investigated under a complex trait analytical framework. Metagenomic profiles of the feed substrates, and comparisons between the Formulated-fed and Sterile-fed groups, were integrated in order to clarify the importance of feed-derived biotic factors in shaping gut communities. Potential independent and feed-specific impacts of gut microbiome structure, taxonomic identity, and inferable function on the development of larval production traits were then assessed.

## 2. Materials and Methods

### 2.1. Larval Rearing, Sample Collection, and Gut Excision

All larvae were collected from experimental colonies established and reared under standard commercial practices at AgriProtein Holdings Ltd. (Cape Town, South Africa). The recently established captive cohort (F2), sourced from Grahamstown, Western Cape (33.3106° S, 26.5256° E), is denoted as the ‘wild’ group. The second genetic background, termed the ‘factory’ group, is represented by a long-standing colony (F62) originally derived from a BSF population in Durban, KwaZulu-Natal (29.8587° S, 31.0218° E), and mass reared under standard commercial conditions. Neonates from both genetic backgrounds were reared on a high-fibre starter feed (prepared by AgriProtein) for four days prior to commencement of this study. Experimental diets included a standard grain-based feed, a mix of brewer’s spent grain and a plant-based sweetener, and a vegetable waste stream, referred to as Formulated, Brewer’s, and Organic diets, respectively. These feeds were selected as they represent common dietary options within the BSF mass-rearing industry. Unpublished proximate analysis of crude compound fractions finds that the relative (%) protein:fat:carbohydrate levels in the feeds are as follows: Formulated—15.3:2.7:66.8; Organic—17.6:9.4:45.2; Brewer’s—16.4:9.2:42.7. Additionally, a feed with minimal microbial contamination was generated by autoclaving the Formulated diet at 121 °C for 20 min; this is referred to in the text as the Sterile feed. This diet was intended to serve as a control for potential influences of feed-borne microbial populations on the dynamics of gut microbiome assembly.

In a factorial experimental design, 200 four-day old larvae of each genetic cohort were placed in 105 × 105 × 145 mm rearing vessels with 336 g of each experimental feed substrate, accounting for a feeding rate of 120 mg/larva/day for each diet without replacement over the duration of the experiment. Larvae were reared in an incubator at 30 °C and 64% relative humidity. All experimental groups were run in triplicate and maintained in parallel. In order to ensure the capture of late instar, non-prepupal, larvae, six white, and actively feeding individuals were sampled from each rearing vessel when roughly 10–25% of larvae showed prepupal characteristics, as per Bruno et al. [35]. Sampled larvae were rinsed with 70% ethanol and distilled water to remove excess feed substrate and then stored individually in 1.5 mL Eppendorf tubes in 100% ethanol at 4 °C. Additionally, feed substrate from each rearing vessel was collected in 15 mL centrifuge tubes and stored at −80 °C.

The remaining larvae were weighed in batches of 30, in triplicate, for the estimation of average larval weight per experimental group and replicate. Individuals from each experimental group and replicate were then pooled in 15 mL centrifuge tubes and dispatched to Quantum Analytical Services (Malmesbury, South Africa) for proximate analysis. Briefly, the nitrogen content of larvae was estimated by using the Dumas method [40], which relies on detection of N_2_ gas emissions following sample combustion under oxygen-rich conditions [41]. Material protein content was then estimated by scaling total nitrogen content by a factor of 6.25, as is deemed appropriate for insect tissue samples [42]. Crude larval fat content was gravimetrically analysed by comparing sample weight before and after a high temperature petroleum extraction [43] performed using the ANKOM XT15 Extractor system.

Larval gut tissue was excised from all collected larvae under standard sterile technique conditions in a laminar flow cabinet. Whole larvae were washed twice briefly in a 10% bleach solution, followed by a rinsing with 70% ethanol. Using an aseptic technique, surgical scissors and tweezers (submerged in 100% ethanol and flame sterilised prior to use) were used to remove the head and final abdominal tergal plate. Larvae were then sectioned horizontally along the spiracles with scissors and teased open by using tweezers. A second pair of tweezers was then used to grip and remove the entire digestive tract bundle, keeping the foregut, midgut, and hindgut intact while removing the associated fat body. Removed gut tissue was fixed in 100% ethanol and stored at −20 °C prior to DNA extraction. All excised gut samples were stored for less than 48 h.

### 2.2. DNA Extraction and NGS Library Preparation

Genomic DNA (gDNA) was extracted from total larval gut samples using the RTP Bacteria Mini Kit (Stratec, Birkenfeldcity, Germany) reagents according to the manufacturer’s instructions. Due to incompatibility with this extraction protocol, feed substrate gDNA was extracted using the *Quick*-DNA™ Fecal/Soil Microbe Miniprep Kit (Zymo Research, Irvine, CA, USA) with a sample input amount of 0.25 g. Sample DNA concentrations were quantified using fluorometry with the Qubit 1× dsDNA HS assay kit (Thermo Fisher Scientific, Waltham, MA, USA), and sample purity was assessed on a NanoDrop^®^ ND-1000 (Thermo Fisher Scientific, Waltham, MA, USA). For the larval samples, all six individual extractions for each experimental group and replicate were normalised to equimolar concentrations based on Qubit assay results and pooled equitably, yielding a single composite larval gDNA sample per experimental replicate. Feed substrate samples were not pooled. A DNA Extended Range LabChip^®^ (PerkinElmer, Waltham, MA, USA) and Genomic DNA Reagent Kit (PerkinElmer, Waltham, MA, USA) was used to validate the quality of gDNA samples by determination of genomic quality scores on the PerkinElmer LabChip GXII Touch. Subsequent 16S hypervariable region amplification, library preparation, and next-generation sequencing (NGS) steps were performed at the Central Analytic Facility (CAF), Stellenbosch University, to the specifications of the manufacturers of the Ion 16S™ Metagenomics Kit (Thermo Fisher Scientific, Waltham, USA).

The Ion 16S™ Metagenomics Kit (Thermo Fisher Scientific) targets a total of seven hypervariable regions for parallel amplification over two reactions with distinct primer sets (V2, V4, and V8 for the first set and V3, V6-7, and V9 for the second set). For each reaction, 2 µL of gDNA was amplified over 25 cycles, after which the presence of amplification products was verified with the X-mark chip and HT DNA NGS 3K Reagent Kit (PerkinElmer, Waltham, USA) run on the LabChip GXII Touch. Following successful amplification, products from both primer pools were combined sample wise, purified using the Agencourt™ AMPure™ XP reagent, eluted to a final volume of 15 µL in nuclease-free water, and subjected to quantification with the Qubit 1× dsDNA HS assay kit.

Library preparation was performed with the NEXTflex™ DNA-Seq Kit (PerkinElmer, Waltham, USA) using 100 ng of pooled PCR product from each sample and following manufacturer protocol. PCR products were end-repaired and purified using the Agencourt™ AMPure™ XP reagent. Barcoded libraries were generated by blunt-end ligation of sequencing adapters and end-repaired PCR products using IonCode™ Barcode Adapter reagents. The resulting libraries were purified with the Agencourt™ AMPure™ XP reagent. Quantification was then performed by using the Ion Universal Library Quantitation Kit (Thermo Fisher Scientific) and by qPCR amplification with the StepOnePlus™ Real-time PCR system (Thermo Fisher Scientific). The LabChip GXII Touch, X-mark chip, and HT DNA NGS 3K Reagent Kit (PerkinElmer) were used to validate library fragment size distribution. Finalised and barcoded libraries were diluted to a concentration of 10 pM, combined in equimolar amounts, and subjected to sequencing template preparation using the Ion 510™ & Ion 520™ & Ion 530™ Chef Kit (Thermo Fisher Scientific). High-throughput, massively parallel sequencing of combined libraries was performed using the Ion S5™ Sequencing Solutions and Sequencing Reagents Kit on the Ion S5 Gene Studio platform following the manufacturer’s protocol. Finally, flow space calibration and BaseCaller analyses were applied in the Torrent Suite Software (version 5.12.0) under default parameters.

### 2.3. Read Processing, Data Filtering, and Normalisation

Raw reads were processed using the 16S Metagenomics Workflow (version 1.1) implemented in the Ion Reporter™ software (Thermo Fisher Scientific). This pipeline trims adapter sequences and subsequently filters out low quality (Phred Q < 20), short (<150 bp) and low abundance (*n* < 10 in all samples) reads. The automated workflow then clusters and assigns operational taxonomic units (OTUs) against the proprietary MicroSEQ^®^ 16S Reference Library (v2013.1) (Thermo Fisher Scientific, Waltham, USA) and open-access Greengenes (v13.5) databases. Initial OTUs were identified using a minimum read-database alignment coverage of 90%, after which genus-level clustering and taxonomic assignment was performed at a similarity threshold of 97%. This procedure was conducted separately for each of the six sequencing library amplicons, and a consensus result for each identified OTUs across all regions was returned. In order to assess whether sufficient bacterial diversity was captured by this workflow, rarefaction curve analysis of the output OTU table was performed in R [44] using the *vegan* package [45].

The full microbiome dataset was then additionally filtered in the MicrobiomeAnalyst webtool [46] to remove further OTUs with low-prevalence (<10%) across all samples. This filtered dataset was used for the calculation of alpha diversity statistics and assessments of relative taxonomic contributions across experimental groups. In the same software, trimmed mean of M-values (TMM) normalisation was applied to the filtered dataset to correct for sparsity and uneven sampling depth without compromising the detectability of differential features between groups [47]. The subsequent filtered and normalised dataset was used for assessments of beta diversity, estimation of linear effect sizes and inference of group-wise metabolic profiles.

### 2.4. Gut Microbiome Diversity and Differentiation Analysis

Alpha (within-sample) diversity measures were calculated using the R-based *phyloseq* [48] and *vegan* packages implemented in MicrobiomeAnalyst. Taxonomic richness was inferred directly from observed OTU counts, while alpha diversity (as a function of both taxon richness and abundance) was assessed using both the Shannon–Weaver statistic [49] and Simpson diversity indices [50]. Homoscedasticity (Levene’s test [51]) and normality (Shapiro–Wilk’s test [52]) of data were assessed before using analysis of variance (ANOVA; *p* < 0.05 significance threshold) to test the influence of feed, larval genetic background and a potential genetics-by-environment interaction on these richness and diversity estimates. A Spearman’s rank correlation test was used to assess whether alpha diversity trends in larval gut samples could be associated with alpha diversity patterns evident in larval feed substrates.

Beta (between-sample) diversity analyses were conducted in R. The statistical significance of group-level microbiome differentiation was assessed by using the *vegan* package’s permutational multivariate analysis of variance (PERMANOVA; permutations = 999) [53], permutational analysis of multivariate dispersion (PERMDISP; permutations = 999) [54], and analysis of similarities (ANOSIM; permutations = 999) [55] applied to a Bray–Curtis dissimilarity index derived from the TMM-normalised OTU abundance tables. In order to determine group comparisons contributing most to the rejections of PERMANOVA H_o_, a pairwise PERMANOVA (permutations = 999) with false discovery rate (FDR) [56] correction for multiple testing was computed using the *RVAideMemoire* [57] package. Between-sample distances were visualised in collapsed ordination space using Non-metric Multidimensional Scaling (NMDS) implemented in *vegan*.

### 2.5. Assessment of Core and Enriched Bacterial Genera

Relative (%) abundance tables were constructed in R to assess the distributions of genus-level taxonomic assignments within each experimental group for the larval metagenomic dataset. Genera with abundances lower than 5% in all samples were considered rare and collectively denoted as ‘Other.’ Putative core genera were identified by applying stringent selection parameters, i.e., greater than 90% prevalence and 5% mean abundance across all samples. Linear discriminant analysis of effect size (LEfSe) [58], designed for the discovery of biologically relevant biomarkers in metagenomic datasets, was applied for the detection of the top 20 taxonomic features (Kruskal–Wallis *p* < 0.05; LDA > 2.0) with association to diet and genetic background grouping variables, respectively. Following the detection of genera associated with host genetic cohort, PERMANOVA tests excluding these taxa were performed (as previously described) to determine whether divergence in larval microbiota profiles by host background was solely described by the differential abundance of these genera.

### 2.6. Relationships between Phenotype and Microbiome Data

Collected phenotypic data (final mass, crude fat content, and crude protein content) were analysed in R. Significance of mean differences in phenotypic measures between experimental groups was assessed using ANOVA (*p* < 0.05). Multiple linear regression modelling in base R was then employed to determine the relationship between phenotypic measures and microbiome data, using the primary and secondary axes of an NMDS (conducted on Bray–Curtis distance matrices) to represent relative compositional differences between samples. These models were expanded to include diet and larval genetic background as covariates, with non-significant features (*p* > 0.05) being eliminated by a backward selection procedure. A variance partitioning feature of the *vegan* package (‘varparts’ function), based on redundancy analysis (RDA), was then utilised to determine the fraction of phenotypic variance that could be uniquely and jointly ascribed to dependent variables in the final linear models. Univariate Spearman’s tests were used to assess the correlation between the relative abundance of singular genera and phenotypic measures. Within each test, samples with zero abundance of the considered taxon were excluded from analysis. Once tests for all microbiota–trait relationships were complete, *p*-values were corrected for multiple testing (FDR correction), and significant relationships were only retained if supported by a final *n* > 12 and an FDR-adjusted *p* < 0.05.

### 2.7. Inference of Gut Functional Profiles

The metabolic potential of gut samples was inferred by using the automated taxonomy-to-phenotype mapping capabilities of the METAGENassist software [59], which integrates information from a custom microbial phenotype database, BAcMap [60], GOLD [61], and other NCBI-based taxonomic databases to derive and compare functional profiles of microbial communities. Analyses were conducted using the TMM-normalised larval microbiome dataset. A heatmap of the relative abundance of metabolic factors between samples was constructed using the ‘Analyze by phenotype’ option. Within this analysis, sample rows were organised into an overlaid dendrogram using the Ward clustering algorithm run on a matrix of pairwise Spearman’s rank correlations. Kruskal–Wallis rank sum testing with FDR correction for *p*-values was then applied to identify metabolic features that show significant differentiation between feed type. Similarly, a Wilcoxon rank sum test with FDR correction was used to assess the two-group hypothesis of significant deviation of metabolic profiles in relation to genetic identity of the larval groups.

## 3. Results

### 3.1. Read and OTU Processing

A total of 26,887,356 reads were generated across the larvae and feed metagenomic libraries constructed for this study (Supplementary Tables S1 and S2). Following sequence trimming and quality filtering steps, 21,121,943 reads were retained, and a further 14,236,865 reads were matched to entries in the MicroSEQ^®^ and Greengenes databases (Tables S1 and S2). Of these, 4,941,674 and 4,426,377 sequences were mapped to 123 and 263 bacterial genera for the larvae and feed libraries, respectively (Tables S1 and S2). Rarefaction curves drawn for both libraries (Supplementary Figure S1) reached saturation, indicating that sampling depth sufficiently captured community diversity. Additional filtering steps reduced larvae and feed datasets to 74 and 99 genera, respectively. It should be noted that the employed mapping procedure recovered the genus *Ruminococcus* twice, assigned as both “Ruminococcus” and “[Ruminococcus]”. Exploration of Krona [62] graphics embedded within this pipeline identified the former OTU to belongs to the Ruminococcaceae family, and the latter to belongs to the Lachnospiraceae family, as is consistent with current paraphyletic structure of Clostridia [63].

### 3.2. Measures of Microbial Alpha Diversity

**Figure 1:**
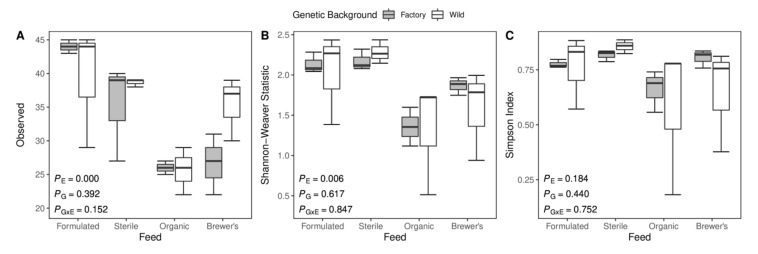
Alpha diversity of larval guts is primarily structured by larval diet. Boxplots of GIT bacterial diversity in terms of (**A**) observable richness, (**B**) Shannon–Weaver diversity, and (**C**) Simpson index values across feed and genetic background partitions of the larval microbiome dataset. Analysis of Variance (ANOVA) *p*-values for testing the hypotheses that mean diversity estimates are significantly influenced by larval feed substrate (*p*_E_), larval genetic background (*p*_G_), and gene-by-environment interaction (*P*_GxE_) are displayed.

The within-sample diversity of microbiomes can be described by a variety of metrics. Here, the observed number of different genera within each group is considered as an indicator of community richness, while the Shannon–Weaver statistic and Simpson index are employed to probe the total diversity of gut microbiomes as a function of both the richness and the relative distribution of different taxa in larval guts. Feed substrate is identified as a substantial driver of within-sample gut microbiota diversity, with significant deviation between groups recovered for observable richness (R^2^_E_ = 0.613, *p*_E_ < 0.001) and Shannon–Weaver (R^2^_E_ = 0.517, *p*_E_ = 0.006) estimates (ANOVA; Figure 1). In general, Formulated-fed and Sterile-fed larvae are suggested to harbour the most diverse microbial populations, with higher mean values across these alpha diversity measures (Figure 1). Correlative tests indicate that patterns of GIT diversity inferred from OTU richness (Spearman’s ρ = 0.732, *p* < 0.001) and Shannon–Weaver estimates (Spearman’s ρ = 0.618, *p* = 0.002) are strongly reflective of bacterial diversity within the feed environment (Supplementary Figure S2). Unlike the Shannon–Weaver statistic, which considers OTU richness and evenness as equally important in determining community diversity, the Simpson index places greater weight on the evenness of taxonomic distributions [64]. As the value of this index is inversely proportional to community evenness, the high values observed indicate that larval gut microbiome communities are skewed towards the dominance of a small number of microbial taxa (Figure 1). Furthermore, by decoupling Simpson diversity between gut communities and exogenous, feed-borne bacterial populations (Spearman’s ρ = 0.332, *p* = 0.113; Figure S2) imply that similar dominance profiles are maintained between larvae irrespective of the effects of diet. Notably, different DNA extraction kits may alter the magnitude and composition of bacterial diversity detected in processed samples [65,66,67], complicating direct comparisons of larval gut and feed substrate diversity. However, diagnostic differences between samples are expected to persist above the noise introduced by using different extraction chemistries [66,68]. Spearman’s tests utilised here are, therefore, expected to produce robust comparisons of larval gut and diet diversity trends as they are concerned only with differences in the relative diversity rank between metagenomic samples.

Little evidence to implicate either genetic background or a feed–background interaction in driving the establishment of gut microbiota diversity is found (*p* > 0.05; ANOVA; Figure 1). All neonates were initially maintained on a high-fibre starter feed before placement on experimental diets (see Methods and Materials); therefore, it is not unexpected that a high degree of bacterial diversity was also observed in the guts of the Sterile-fed larvae.

### 3.3. Microbiome Structure and Differentiation

Permutational multivariate testing yields considerable evidence for compositional differentiation of the larval gut microbiota dataset by feed (PERMANOVA; R^2^_E_ = 0.374, *p* < 0.001) (Table 1). Although the associated effect sizes are small, larval genetic background (R^2^_G_ = 0.078, *p* = 0.009) and the potential interactions between feed and background (R^2^_GxE_ = 0.139, *p* = 0.031) are also recovered as microbiome structuring variables (PERMANOVA; Table 1). Non-significant PERMDISP results for stratification of the dataset by feed (*F*_E_ = 1.424, *p* = 0.248) and larval strain (*F*_G_ = 1.934, *p* = 0.176) reinforce the validity of these findings. Post hoc pairwise tests found microbiota differentiation between all but the comparison of Organic-fed and Brewer’s-fed larvae (Supplementary Table S3). Additional ANOSIM testing also supports that feed substrate drives GIT community differentiation (R = 0.417, *p* = 0.001) but provides little evidence for microbiome divergence by genetic background alone (R_G_ = 0.088; *p* = 0.088). Notably, a great deal of compositional variation in this system remains unexplained by the grouping variables (Residual R^2^ = 0.409) (PERMANOVA; Table 1).

**Figure 2:**
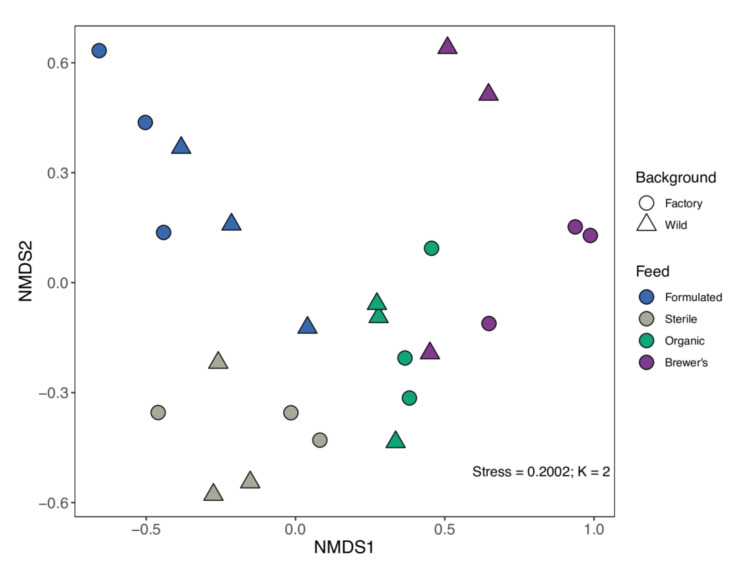
Non-metric Multidimensional Scaling (NMDS) analysis of OTU dataset. Analysis was conducted using two explanatory axes (K = 2) and is described by a final configuration stress-value of 0.2002. Samples are coloured by provisioned experimental diet, with different shapes representing sample genetic identity.

The NMDS analysis employed clearly resolves the effect of dietary variation on microbiome assembly, with samples clustering by feed group along the primary axis (Figure 2). The influence of host genetic background on sample compositionality is, however, less apparent in ordination space (Figure 2). Given that the observed genetic effects are small in comparison to those of the diet (Table 1) and further complicated by interactions with environmental variables, it is understandable that a simple 2D representation of sample dissimilarities may not adequately capture the impact of host genetic background on microbiome structure. Indeed, when NMDS was conducted on diets independently, a more coherent, strain-dependent pattern of microbiota diversity emerges (Supplementary Figure S3).

### 3.4. Production Trait Measures and Relationships to Microbiome Structure

**Figure 3:**
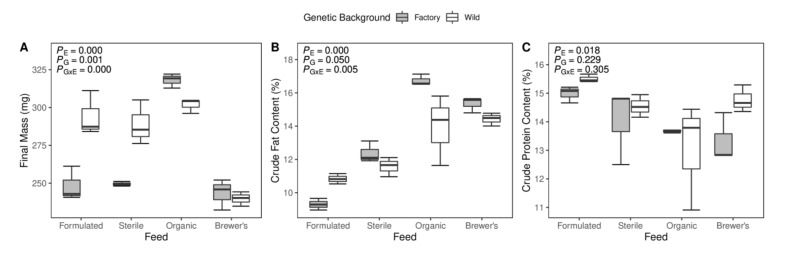
All measured larval production traits are structured by host genetic background or diet. Boxplots of larvae (**A**) final mass, (**B**) crude fat content and (**C**) crude protein content measures stratified by feed and genetic background. Analysis of Variance (ANOVA) *p*-values for testing the hypotheses that trait values are significantly structured by larval feed substrate (*p*_E_), larval genetic background (*p*_G_), and gene-by-environment interactions (*p*_G×E_) are displayed.

Economically important traits related to larval bioconversion efficiency, namely final mass, protein content, and lipid content, were measured across all groups. Inspection of the phenotypic data indicates that larval final mass is significantly structured by the provisioned feed substrate, with Organic-fed specimens showing higher mean weight than their experimental counterparts (Figure 3A). A further interactive effect of feed and genetic background is observed, with diet-dependent divergence in mass between wild and factory larvae (Figure 3A). Similarly, larvae of the Organic and Brewer’s groups show substantially higher end-point fat percentage than those reared on Formulated and Sterile diets (Figure 3B). Again, an interaction between feed and larval strain is suggested by a trend of reduced mean fat content in wild versus cultured larvae (Figure 3B). Only a dietary effect was identified for larval protein content, with members of the Formulated and Organic groups being the most and least protein rich, respectively (Figure 3C).

Linear regression modelling suggests that gut microbiome structure could play a role in the emergence of select larval phenotypes. Using the NMDS axes as a proxy for overarching relative compositional differences between samples, evidence is found for relationships between gut microbiota and the lipid (NMDS1 Adjusted R^2^ = 0.683, *p* < 0.001) and protein (NMDS1 Adjusted R^2^ = 0.235, *p* = 0.010) content of larvae, respectively. However, controlling for the feed variable in multiple linear regression models entirely suppresses the significance of gut structure in determining larval protein content (NMDS1 *p* = 0.110) but retains the relative importance of the microbiome in regulating fat accumulation (NMDS1 *p* = 0.038). In each case, the secondary NMDS axis and larval genetic background variables were removed by backward elimination. Partitioning analysis suggests that little variation in lipid content measures can be ascribed independently to either gut structure (3.9%) or feed substrate (11.8%) and that a great proportion of explanatory variance in the linear model is shared between these variables (64.3%) (Supplementary Figure S4). Additional testing finds no evidence to suggest that divergence in overarching GIT microbiota profiles underlies significant between-group divergence in the larval mass phenotype.

### 3.5. Taxonomic Profiles, Core Genera, and Patterns of Enrichment

**Figure 4:**
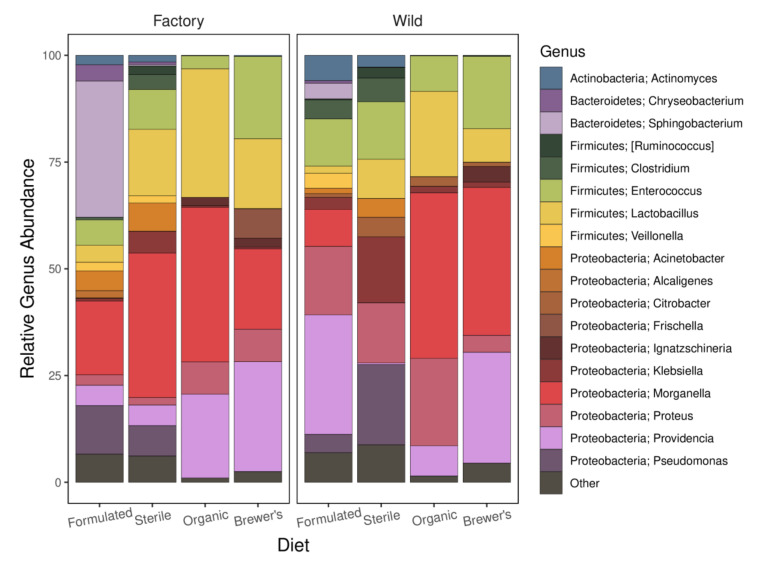
The distribution of bacterial genera within the guts of *H*. *illucens* larvae. Average group-wise relative abundance (%) of genus-level taxonomic assignments to the OTU dataset (*n* = 3) are displayed. Genera with lower than 5% abundance across all samples are classified as Other.

The assessment of relative taxonomic abundance profiles indicates a diverse larval gut microbiome across experimental groups (Figure 4). The majority of mapped bacterial genera belong to the Proteobacteria (48) phylum, with remaining OTUs assigned to members of the Firmicutes (16), Actinobacteria (8), and Bacteroidetes (2) taxa. At an arbitrary 5% mean relative abundance threshold, the *Morganella* (23.5 ± 24.6%), *Providencia* (14.5 ± 16.6%), *Lactobacillus* (13.1 ± 11%), *Enterococcus* (10.9 ± 7.0%), and *Proteus* (9.3 ± 9.9%) genera are observed to be both dominant and ubiquitous across all cohorts (Supplementary Table S4). These taxa may, therefore, be regarded as core members of the larval GIT community, where together they account for 73.3% of all genus-level mapped reads for the larvae sequencing library. Correlation testing between the larvae and feed relative abundance datasets suggests that the dominance of *Morganella* (Spearman’s ρ = 0.569, FDR-adjusted *p* = 0.015) and *Lactobacillus* (Spearman’s ρ = 0.591, FDR-adjusted *p* = 0.014) in larval guts may, in part, be explained by the prevalence and abundance of these bacteria in feed substrates (Supplementary Figure S5).

**Figure 5:**
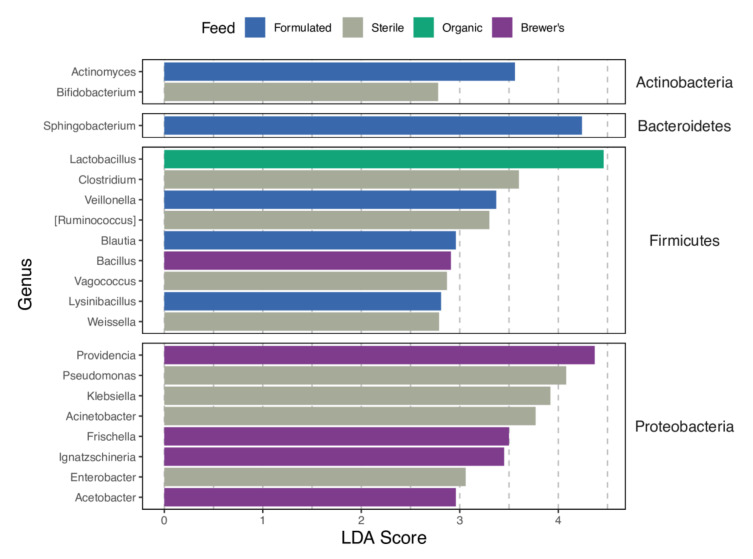
Bacterial genera that show diet-dependent patterns of enrichment following Linear Discriminant Analysis of Effect Size (LEfSe; FDR adjusted *p* < 0.05; LDA score > 2.0) testing. The top 20 associations in terms of LDA score are displayed, with bar colours identifying the specific feed substrate correlated with significantly increased taxon abundance. The test is conducted without consideration of the larval genetic background variable.

Diet-dependent patterns of enrichment are detected for numerous bacterial taxa (Figure 5), many of which are also highly abundant in larval gut microbiomes (Figure 4). Provision of both the Formulated and Sterile diets is associated with an increased abundance of genera spanning the Actinobacteria, Firmicutes, and Proteobacteria phyla, while the Formulated diet alone is also described by an increased presence in a Bacteroidetes member, *Sphingobacterium*. In contrast, the Brewer’s and Organic diets show a narrower phylogenetic range of distinguishing bacterial genera. Brewer’s feed was predominantly associated with increased levels of proteobacteria, while Organic feed was differentiated only by the elevated abundance of *Lactobacillus* (Firmicutes). Significant correlations between taxon abundance in the feed substrate and intestinal system (Figure S5) suggest that divergent environmental microbial profiles may underlie detection of diet-specific enrichment patterns for many gut microbiome members. The *Citrobacter* genus, which shows no evidence for this proposed feed–gut abundance relationship (FDR-adjusted *p* = 0.255), is recovered as a distinguishing biomarker in wild versus factory BSF microbiomes (LDA score = 3.35; FDR-adjusted *p* < 0.001). Interestingly, the exclusion of this genus does not depress the significance of host genetic background in driving microbiome structure (R^2^_G_ = 0.070, *p* = 0.020; R^2^_GxE_ = 0.140, *p* = 0.036; PERMANOVA; Supplementary Table S5).

Stringent univariate Spearman’s correlation tests implicate the *Lactobacillus* genus in driving fat accumulation rate in larvae (Spearman’s ρ = 0.750, FDR-adjusted *p* < 0.001; *n* = 23). Similarly, the abundance of the *Actinomyces* genus, which is entirely absent from the gut communities of Organic-fed larvae, is associated with differences in the final mass of samples across the Formulated, Sterile, and Brewer’s diets (Spearman’s ρ = 0.847, FDR-adjusted *p* < 0.001; *n* = 16).

### 3.6. Inference of Gut Functional Profiles

**Figure 6:**
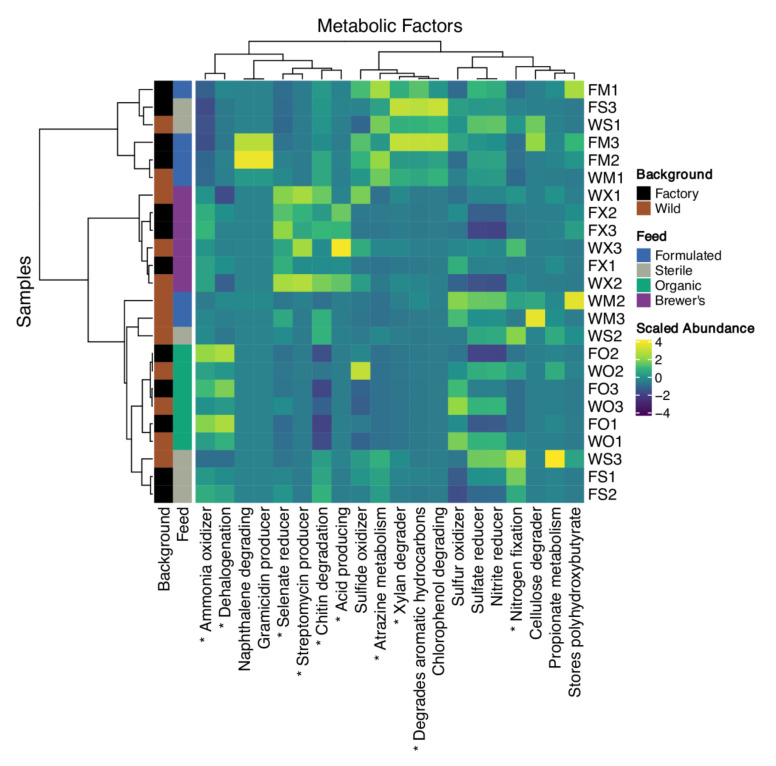
Heatmap visualisation of inferred metabolic potential of larval gut microbiomes. Hierarchical clustering of samples and metabolic factors (row-wise normalisation) by Ward’s method applied to Spearman’s rank correlation coefficient distance (1-ρ) matrices. Tree tips are coloured by larval genetic background and experimental feed substrate. Metabolic factors that show significant (FDR-adjusted *p* < 0.05) abundance differences between feed groups are denoted by an asterisk (‘*’). Samples are alphanumerically encoded. F—Factory; W—Wild; M—Formulated; O—Organic; S—Sterile; X—Brewer’s. Sample numbers refer to each pooled experimental replicate.

The METAGENassist software was employed to explore mechanistic implications of GIT microbiota diversity, inferring a global putative functional profile of 21 metabolic factors from the 74 genus-level OTU assignments made against the larval 16S metagenomic library. Similarity-based clustering regarding the relative abundance of these factors largely reflects feed substrate partitions of this dataset, with consistent distinction of the gut functional profiles of Brewer’s-fed and Organic-fed larvae (Figure 6). Variability in the metabolic potential of Formulated and Sterile gut bacterial communities is pronounced, with samples tending to co-cluster at various positions throughout the sample dendrogram (Figure 6).

Ten metabolic phenotypes show significant differentiation between these diet groups (FDR-corrected *p* < 0.05; Figure 6; Supplementary Table S6). The potential for degradation of atrazine, aromatic compounds, and xylans seems to be substantially enriched in the guts of larvae fed with Formulated and Sterile diets, while nitrogen fixation capacity is observed to be a dominant feature in GIT microbial communities of the latter feed only. Alternatively, high abundances of streptomycin producing, acid producing, and selenate reducing bacteria characterises the Brewer’s group. Ammonia oxidisation capabilities seem to follow a gradient from notable upregulation in the Organic group to marked downregulation in the guts of Formulated and Sterile-fed larvae. A similar pattern is noted for chitinase activity, where the Brewer’s-fed group shows the greatest abundance of this metabolic category while the Organic group is comparatively depleted of chitin-degrading bacteria. Finally, the potential for dehalogenation metabolism is observed to be uniquely predominant in the intestinal microbiota of Organic-fed larvae. Neither clustering relationships nor patterns of factor enrichment implicate larval genetic background as a clear driver of gut metabolic function (Figure 6).

## 4. Discussion

A comprehensive understanding of larval microbiota dynamics is an essential precursor for the identification of host–microbiome relationships with reproducible and exploitable effects on marketable BSF phenotypes [16]. Herein, a 16S rDNA profiling approach was used to explore the broad-scale effects of feed, host genetic background, and putative gene-by-environment interactions on variation in this internalised biological system. Subsequently, the implications of gut community structure and feed-mediated microbial enrichment patterns on the development of commercially important larval production traits (final larval mass, fat content, and protein content) were assessed.

### 4.1. Gut Microbiome Diversity Is Driven by Feed and Host Genetics

Although diet is known to markedly influence larval gut microbiome diversity in BSF [35,69,70], knowledge of the differential effects associated with the provision of industry-relevant feed substrates is sparse. Compared to the protein-rich and fat-rich Organic and Brewer’s feeds, the high-fibre, grain-based Formulated and Sterile diets are associated with significantly greater bacterial diversity in terms of richness and Shannon-Weaver estimates (Figure 1). Similarly, structuring effects of diet on larval gut microbiome compositionality is well-resolved in NMDS ordination space (Figure 2), where primary separation occurs between samples reared on high-carbohydrate (Formulated and Sterile) and high-protein (Organic and Brewer’s) diets, with some secondary deviation within these feed categories. Accordingly, statistical tests attribute a considerable portion of total microbiome variance to differences in feed substrate (R^2^_E_ = 0.374; Table 1), with pairwise divergence detected for all but the comparison between Organic-fed and Brewer’s-fed larvae (Table S3).

These findings are consistent with mounting evidence from literature that the nutritional content of feed, particularly the fat-to-carbohydrate (F:C) and protein-to-carbohydrate (P:C) ratio, plays a sizeable role in shaping gut microbial communities [71,72,73,74,75]. For example, in mouse and *Drosophila* models, gut microbiome alpha diversity is significantly suppressed as the amount of dietary protein and fat increases relative to carbohydrate levels and is often accompanied by large shifts in dominant GIT bacterial taxa between experimental groups [76,77]. However, the exact relationship between these nutritional features and gut microbiome diversity is less clear in BSF, where the directionalities of microbiota diversity responses to stark shifts in dietary F:C and P:C ratios are not necessarily reproduced between studies [35,69,70]. To an extent, this implies that additional feed-specific factors may influence larval gut community dynamics. In this study, significant coupling of bacterial diversity estimates between the diet and intestinal environments (Figure S2) is observed. Furthermore, it is recognised that provision of the Formulated and Sterile feeds, which differ only in microbial burden, imposes divergent larval microbiome structure (Table S3). Jiang et al. [37] observed similar interactions at the interface between feed-borne and gut bacterial populations in BSF rearing systems, finding that taxonomic profiles of larval guts progressively converged towards those of the diet over time. Moreover, Klammsteiner et al. [70] describe resilience of established BSF microbiomes to diet-induced shifts when larvae are reared on microbially limited diets. Taken together, these findings emphasise that interactions between exogenous bacterial populations and resident gut bacteria could play an important but poorly understood role in establishing the larval GIT communities. It is, therefore, apparent that contemporary understanding of the mechanisms that underlie the interplay between environmental factors and gut microbiome dynamics in BSF may be largely oversimplified and open to future refinement.

Recent studies have instigated a paradigm shift regarding our understanding of the fundamental principles that govern the diversity of complex gut ecosystems, advocating for a more pronounced influence of host genetics than previously thought [78]. While these effects are most evident at evolutionary scales [79,80], patterns of co-segregation between host genetic and microbiome factors in human, murine, and swine models highlight that substantial individual-level heritability in animal GIT microbial landscapes can be anticipated [81,82,83,84,85]. Herein, it is expected that the studied larval cohorts are genetically distinct as BSF populations experience large decreases in genetic variation and rapid generational differentiation in closed mass-rearing systems [21]. Given that potential confounding factors (e.g., differences in rearing locale and conditions, feed preparation, and neonate starter feeds) are controlled for between groups in this experiment, the significant fraction of gut microbiome variation explained by larval genetic background (R^2^_G_ = 0.078; Table 1) and background-by-environment interaction (R^2^_G×E_ = 0.139; Table 1) likely reflects genetic divergence between the wild (F2) and long-standing factory (F62) cohorts at loci that govern microbial dynamics. The persistence of these relationships upon removal of the *Citrobacter* genus (Table S5), which showed evidence for differential enrichment in wild larvae, implies that host genetic control of gut microbes may be exerted at broader, community-wide levels in this species.

Quantitative genetics studies have described putative determinants underlying patterns of microbiome heritability, commonly ascribing host-mediated control of gut community assemblages to genes of the immune system [29,30,85,86] and metabolic [87] pathways. Furthermore, it has been illustrated that microbiome–host dynamics may be highly complex and governed by a variety of polygenic, pleiotropic, or even environment-interactive genetic mechanisms [86,88,89,90]. Drawing from immunological research in the literature, it is evident that antimicrobial peptide genes may drive host–microbe interactions to a large extent in BSF [91,92,93,94,95]. Consolidating this with the understanding that diet distinctly impacts expression of these factors [94], it is proposed that a targeted study of this diverse gene set may explain a substantive portion of the genetic and gene-by-environment variance implicated in shaping larval GIT communities. As genomic resources become increasingly available [22,94], the utilisation of quantitative genome-metagenome mapping approaches should provide greater resolution of the specific genetic factors that govern BSF microbiota dynamics.

Notably, a sizeable fraction of variance in gut microbiome compositionality remains unexplained by the experimental design (R^2^ = 0.409; Table 1). Stochastic processes governing the colonisation and stabilisation of gut communities are expected to contribute to this residual variation [96]. For example, ecological drift (i.e., random shifts in the prevalence of different microbial species over time) can cause pronounced differentiation between microbiomes, particularly when many taxa with low relative abundances are present [97,98]. The guts of Sterile and Formulated larvae are likely to be prone to these effects, as they harbour a high diversity of rare microbial taxa (Figure 1). It is, therefore, likely that ecological drift has driven the pronounced compositional (Figure 2) and functional (Figure 6) variation that is especially evident in these groups. However, unsampled deterministic factors may also confound between-group variation in this system. For example, uncharacterised infection with endosymbionts (e.g., *Wolbachia*) or sex-dependent feeding behaviours would be expected to drive divergent microbiome assembly in insect hosts [99,100]. Furthermore, a considerable portion of such residual variation may reflect between-group cohesion as a function of exposure to identical neonate starter feeds or may be driven by global microbiota responses to changes in compositional features of the feed substrate (e.g., moisture content, carbon level, and sulphur level) elicited by general larval action over the rearing cycle [37].

### 4.2. Overarching Gut Microbiome Structure Is Related to Larval Fat Content

The seminal work of Ross et al. [101] illustrates that gut microbiomes, alongside genetic and environmental factors, may have considerable and quantifiable effects on the emergence of complex animal traits. In particular, they observe that integration of microbiota profile data has potential to vastly improve our understanding of how metabolism-related disease states and host phenotypes emerge. Further research in humans has established that body mass index and blood lipid levels may be substantially influenced by microbiome compositionality, irrespective of the confounding effects of age, sex, and individual genotype [102]. These findings are recapitulated in swine models, where microbiota profiles have strong predictive potential for various mass-related traits, including feeding efficiency and final carcass weight [84,103]. Given that the animal GIT microbiome itself behaves as a quantitative phenotype, Camarinha-Silva et al. [84] postulated that there is a precedent for accelerated improvement of valuable but complex livestock production traits via tandem, selective optimisation of host genotype, and microbiota. It is, therefore, unsurprising that growing recognition of this deterministic role for the microbiome in phenotype development has resulted in the consideration of expanded, holobiont trait models that incorporate the independent effects of host genotype, the environment, gut microbiota, and their relevant interactions [104].

Although less commonly explored, similar microbiome–phenotype relationships are evident in insect species. For instance, in the Glanville fritillary butterfly, it seems that complex trait phenotypes, such as larval growth rate, are predominantly influenced by GIT community structure, even when accounting for the effects of larval diet [105]. Furthermore, Chaston et al. [106] comprehensively defined a significant relationship between the nutritional index and enteric microbiota composition of *Drosophila*, uncovering additional evidence for causal host–microbiome interplay in the emergence of these traits. The findings support a comparable link between overall microbiome structure and the rate of fat accumulation in BSF larvae, where microbiome compositionality independently explains at least 3.9% of this trait’s variance. While the magnitude of this explanatory variance is in line with other studies [102], the indistinguishable effects of feed (i.e., multicollinearity between the dependent variables) may obscure the true effect size of the microbiota in driving the development of this phenotype. It is, therefore, proposed that increased sample size, in the absence of a structured study design, may provide more statistical power for the detection of significant microbiome–trait relationships in future investigations. Nonetheless, in light of the complexity of microbiota assembly in BSF, it remains apparent that the identification of optimal microbiome configurations that maximise trait development requires a contextual understanding of how the effects of host genetics, the environment, and the microbiome coalesce to drive economically important phenotypes in this species.

### 4.3. The Implications of Taxonomic and Metabolic Gut Microbiome Profiles

The analysis of taxonomic OTU classifications highlights that differential feed provision may have implications for host development by driving marked turnover in bacterial community identity and metabolic functionality. Considering the Organic-fed larvae, which display the most favourable mass and fat accumulation rates (Figure 3A,B), significant enrichment for members of the *Lactobacillus* genus is observed (Figure 5). Furthermore, correlative tests strongly support a relationship between the abundance of this taxon and larval fat content across all diets. In line with this, inoculation of larval feed substrates with *Lactobacillus buchneri* has been found to improve the relative fat percentage, protein content, and feed conversion efficiency of BSF [33]. Storelli et al. [107] expanded upon this potential relationship in *Drosophila*, showing that *Lactobacillus plantarum* promotes larval growth by stimulating absorption of dietary protein in the gut. The inferred metabolic profile of GIT communities in the Organic larvae is otherwise balanced, with only ammonia oxidation and dehalogenation pathways showing signs of relative upregulation (Figure 6). While enrichment of the former metabolic category may suggest improved clearance of potentially toxic protein metabolism by-products in the gut [108], the biological significance of enhanced dehalogenation metabolism is unclear.

Rearing larvae on the Formulated and Sterile diets is associated with the enrichment of Actinobacteria, Bacteroidetes, and Firmicutes taxa (Figure 5). Among this diverse spectrum of bacteria, numerous genera are likely to contribute pectinases, cellulases, and xylanases that enhance energy extraction from these recalcitrant, fibre-rich diets [27,109,110]. This functionality may be reflected in significant upregulation of the xylan and aromatic hydrocarbon degradative pathways in the guts of these larvae (Figure 6). Despite being entirely absent from the GIT communities of larvae fed with the Organic diet, the abundance of *Actinomyces* is found to be significantly associated with a divergence in mass gain between the remaining feed substrates. While these taxa may support host nutrition via the production of vitamins and degradation of complex biopolymers, it is also likely that they impact host developmental efficiency by modulating pathogen load in the gut [111].

Alternatively, proteobacterial bloom, evident within the GIT communities of larvae reared on the Sterile and Brewer’s feeds (Figure 5), may bear negative consequences for host fitness. Bruno et al. [35] found that significantly diminished larval growth rate in BSF can be attributed to diet-specific enrichment of these bacteria, particularly the *Providencia* genus. They propose that gut dysbiosis, which describes unfavourable compositional and functional alterations of GIT communities [112], may mediate this impact on larval health. Mechanistically, this is congruent with the pathogenesis of proteobacterial genera, many of which may drive persistent community imbalances and reduce the metabolic efficiency of the gut system [113]. Here, a comparable but non-significant trend of reduced growth rate for the Brewer’s-fed larvae is noted (Figure 3). Interestingly, increased abundance of these taxa in the Brewer’s group is accompanied by enrichment for antibiotic and chitin-degradative activities (Figure 6), which have the potential to markedly adjust microbiota balance [114] and increase the host’s susceptibility to opportunistic infection [115].

Despite showing signatures of differential enrichment between feeds, conservative selection criteria (found in 90% of the samples and >5% mean abundance) recover both *Lactobacillus* and *Providencia* as core members of the larval gut microbiome. Their potential relationship to larval growth characteristics, therefore, resonates with the notion that balancing core gut members is a crucial determinant of feeding efficiency in BSF [22]. Nevertheless, three core bacterial genera that display comparatively stable abundance across larval guts are identified. Of these, *Enterococcus* is often recovered as a symbiont of insects [116,117] and is the only genus that is reproducibly identified as a core member of the BSF gut across other studies [36,37,38,70]. The ubiquity of this taxon could be explained by functional dependence in the intestinal system, where *Enterococcus* species may contribute to nutritional provisioning [118] and the host’s repertoire of antimicrobial defenses [119]. Further evidence indicates that the prevalence of this genus may also be a function of vertical transmission [120]. While both *Morganella* and *Proteus* are considered commensals in animal gut systems [121], understanding their role in these symbioses is largely unresolved. Together with *Providencia*, all three of these closely related genera are implicated as human pathogens [122]. The presence of the *Morganella* genus, in particular, has been associated with significantly increased mortality in fruit flies [123]. The consistently high abundance of these bacteria in larvae, therefore, raises the question of whether they are an important biological feature of optimum gut communities or if they are merely tolerated by BSF larvae as they pass through the digestive tract. Indeed, similar questions may be raised regarding the numerous genera that show abundance correlations between the feed and gut environment (Figure S5).

## 5. Conclusions

The black soldier fly, *Hermetia illucens*, is a promising candidate insect species for mass-rearing programmes that aim to address issues of food system sustainability via large-scale bioremediation, nutrient upcycling, and the provision of alternative protein sources for human and animal consumption. While prior studies have highlighted the potential for rapid improvements in larval feeding efficiencies and mass gain through manipulation of BSF gut microbiota, a lack of knowledge regarding the drivers of these bacterial systems poses a challenge to the identification of robust and reproducibly exploitable microbiome–phenotype relationships. In the current study, various statistical approaches indicate that the *H*. *illucens* microbiome is significantly shaped by both host genetic and environmental factors, akin to a classical complex, multifactorial trait. Furthermore, evidence suggests that resultant enteric community compositionality, independent of feed and host genetic background, is significantly associated with the rate of fat accumulation in larvae. Examination of feed-specific bacterial enrichment patterns and their coincidence with phenotypic variation highlights that additional interactions between the environment and the microbiome have substantial impacts on larval developmental traits. Together, these factors affirm microbiome-driven improvement of host phenotypes with economic or bioremedial significance but illustrate the importance of context-specific elucidation of gut microbiota dynamics.

## Figures and Tables

**Table 1:** Larval gut microbiomes are structured by diet, host genetic background, and an interaction between these factors. Results of a permutational multivariate analysis of variance (PERMANOVA; 999 permutations) performed on a genus-level Bray–Curtis matrix of larvae microbiome data. Df—degrees of freedom; SS—model sum of squares. Statistical significance is considered at an α = 0.05 threshold.

Data Partition	Df	SS	R^2^	Pseudo-*F*	*p*
Background (G)	1	0.357	0.078	3.053	0.009
Feed (E)	3	1.714	0.374	4.885	<0.001
Background × Feed (G × E)	3	0.637	0.139	1.815	0.031
Residual	16	1.872	0.409		
Total	23	4.580	1.000		

## Data Availability

The 16S rDNA NGS data produced in this study are available in the National Centre for Biotechnology Information’s (NCBI) Sequence Read Archive (SRA) repository, under BioProject ID PRJNA683575 (https://www.ncbi.nlm.nih.gov/bioproject/PRJNA683575). All other data not presented in the main text are displayed in Supplementary Figures S1–S5 and Supplementary Tables S1–S6.

## Supplementary Materials

The following are available online at https://www.mdpi.com/article/10.3390/insects12121082/s1, Figure S1: Rarefaction curves for samples from the (A) larval and (B) feed 16S metagenomic sequencing libraries. Within libraries, experimental groups are coded by letters: W—Wild; F—Factory; M—Formulated; O—Organic; S—Sterile; X—Molasses. Numbers indicate the rearing replicate, Figure S2: Alpha diversity trends are coupled between feed-borne and larval-gut bacterial populations. Spearman’s ρ and associated *P*-values for testing diversity correlations in terms of (A) observable richness, (B) Shannon–Weaver statistics, and (C) Simpson diversity indices are displayed, Figure S3: Subtle host-genetic effects on microbiome assembly are evident when stratifying microbiome data by feed group. Ordination-space visualisation of Non-metric Dimensional Scaling (NMDS) analysis of samples within the (A) Formulated, (B) Organic, (C) Sterile, and (D) Brewer’s diet groups. Larval genetic background is indicated by datapoint shape, Figure S4: Bacterial genera whose abundance is significantly correlated between the larval gut system and feed substrate. The red, dashed line indicates the log-transformed α = 0.05 significance threshold. Taxa with significant feed-gut abundance relationships following FDR-adjustment of Spearman’s *P*-values are labelled and coloured in black. Some genera with significant correlations (*Acidomonas*, *Brucella*, *Frischella*, *Mycoplana*, *Salmonella*, and *Trabulsiella*) are excluded as *P*-values of zero are not amenable to log transformation, Figure S5: Variance partitioning analysis describes effects of diet and gut microbiome composition on larval fat content. Comparative larval microflora structures are represented by NMDS axis 1 (see Figure 2). Independent effects (‘Diet’ and ‘Microbiome’) are displayed alongside indistinguishable joint effects (‘Diet | Microbiome’) of these variables on the trait in question, Table S1: Read generation, retention, and mapping statistics for the larvae 16S metagenomic library, Table S2: Read generation, retention, and mapping statistics for the feed 16S metagenomic library, Table S3: Pairwise differentiation in larval gut microbiome profiles is observed between feed groups, Table S4: Prevalent and abundant genera across larval gut microbiomes, Table S5: Larval gut metabolic features with significant (FDR-adjusted *p*-value < 0.05) differentiation by feed type, Table S6: Host genetic background is retained as a driver of microbiome structure upon exclusion of the *Citrobacter* genus.
