# Supplementary figures and images for: Feed and Host Genetics Drive Microbiome Diversity with Resultant Consequences for Production Traits in Mass-Reared Black Soldier Fly (Hermetia illucens) Larvae
Source: Insects. 2021 Dec 1;12(12):1082. doi: 10.3390/insects12121082 (PMC8706267; doi:10.3390/insects12121082)

**A**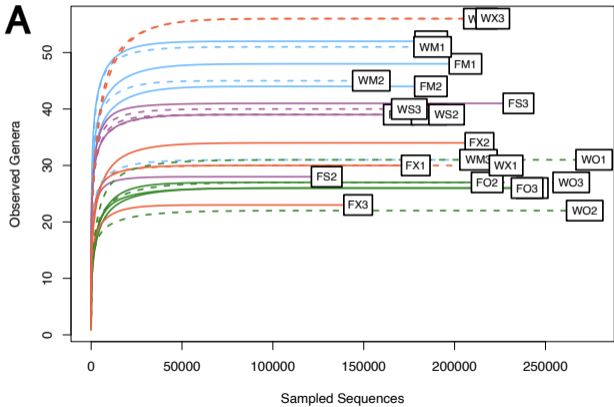**B**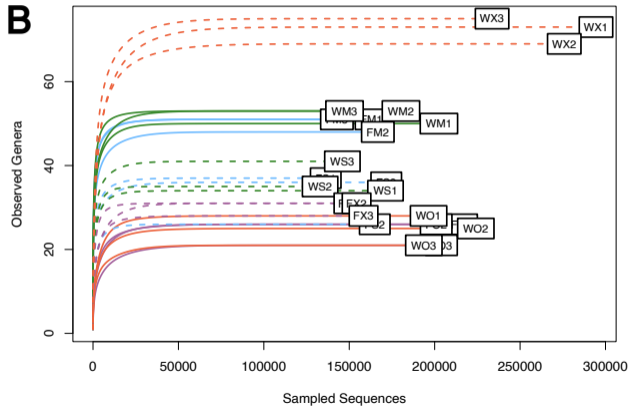

Supplement: Supplementary file 1 [file insects-12-01082-s001.zip › Suppl Files/Figure S1.pdf]

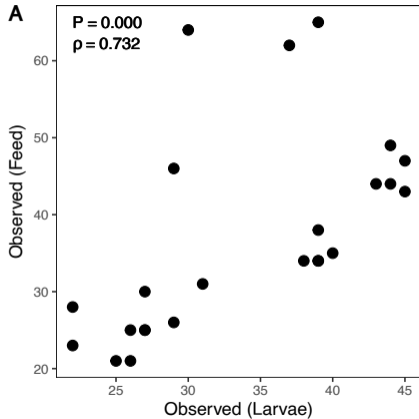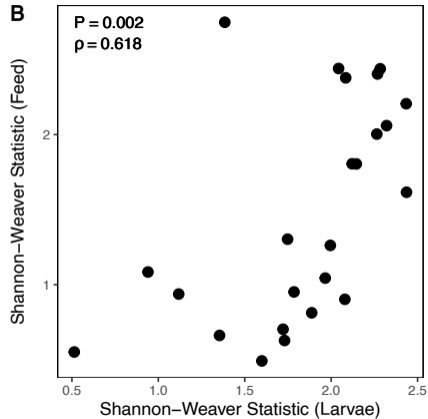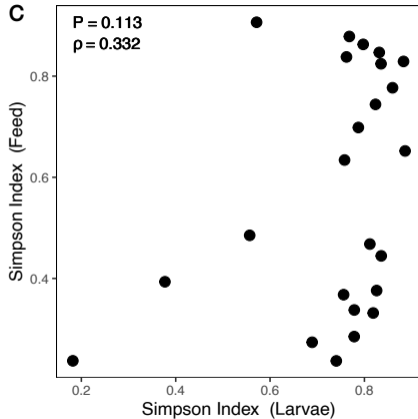

Supplement: Supplementary file 1 [file insects-12-01082-s001.zip › Suppl Files/Figure S2.pdf]

## Background

● Factory ▲ Wild

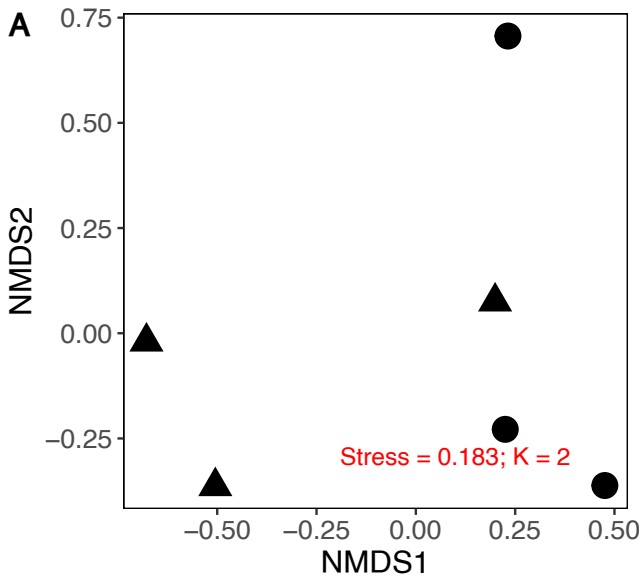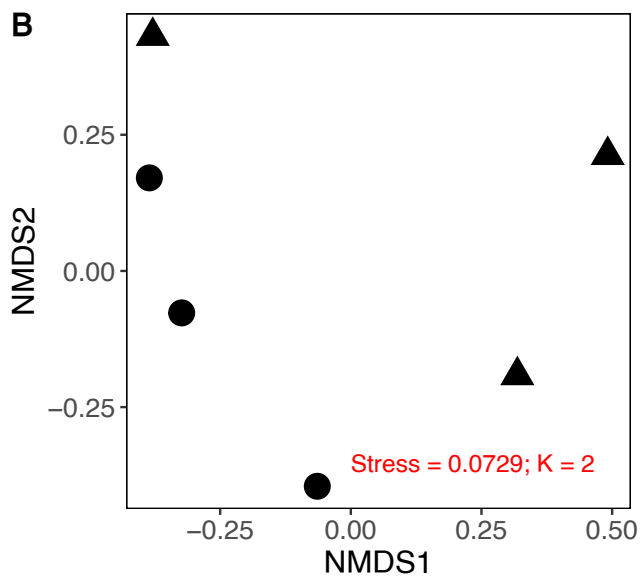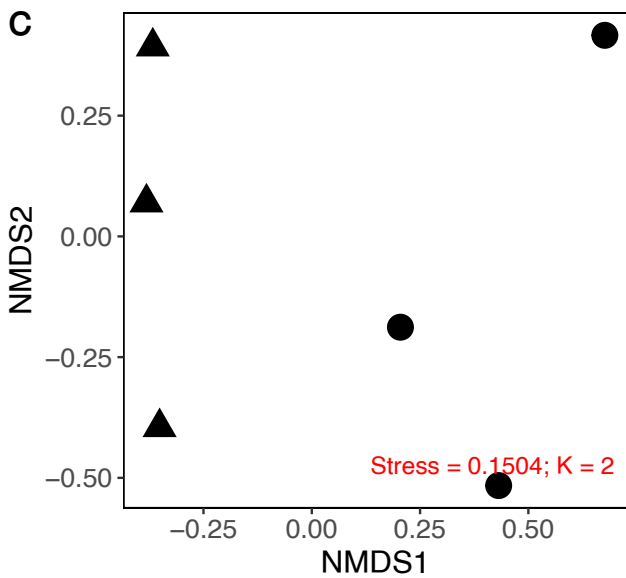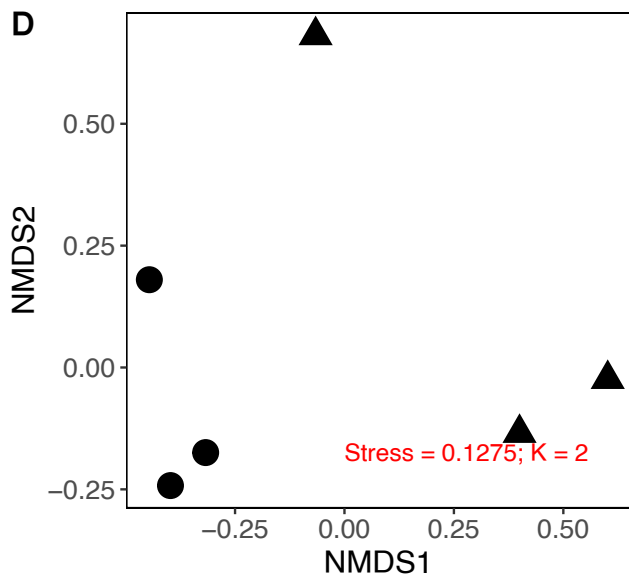

Supplement: Supplementary file 1 [file insects-12-01082-s001.zip › Suppl Files/Figure S3.pdf]

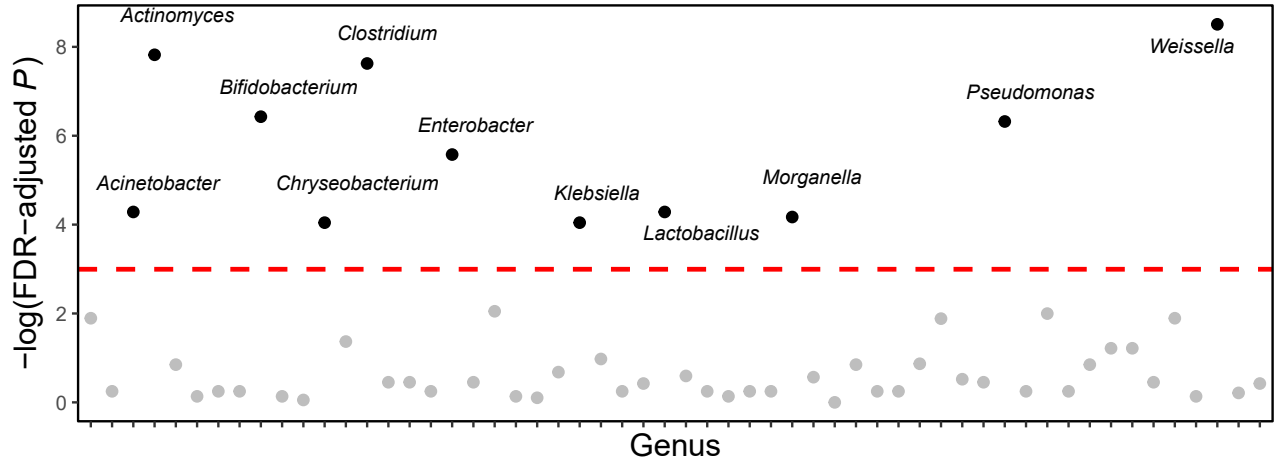

Supplement: Supplementary file 1 [file insects-12-01082-s001.zip › Suppl Files/Figure S4.pdf]

Diet | Microbiome (64.3%)

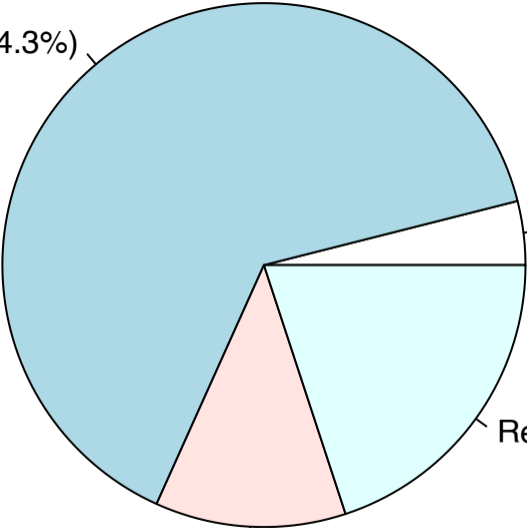

Microbiome (3.9%)

Residual (19.9%)

Diet (11.8%)

Supplement: Supplementary file 1 [file insects-12-01082-s001.zip › Suppl Files/Figure S5.pdf]
